# Sexually Transmitted Diseases among Saudi Women: Knowledge and Misconceptions

**DOI:** 10.3390/ijerph20064858

**Published:** 2023-03-09

**Authors:** Israa Abdullah Malli, Basmah Abdullah Kabli, Lujain Ali Alhakami

**Affiliations:** 1 Department of Basic Medical Sciences, College of Medicine, King Saud bin Abdulaziz University for Health Sciences, Jeddah 21423, Saudi Arabia; 2 King Abdullah International Medical Research Center, Jeddah 21423, Saudi Arabia; 3 Ministry of National Guard—Health Affairs, Jeddah 21423, Saudi Arabia

**Keywords:** knowledge, misconceptions, female, sexual, transmitted, STDs-KQ, college

## Abstract

The rate of sexually transmitted diseases is increasing globally. Thus, this study aimed to examine the Al akami female community’s knowledge about the nature of sexually transmitted diseases and their associated factors. The STDs-Knowledge Questionnaire (STDs-KQ) was utilized to collect data from the female community (355) in Jeddah, Saudi Arabia. The data were analyzed using JMP Statistics for Windows, version 15. The significance level was set at 0.05. The study reported that participants had a relatively low understanding of STDs in acquisition, protection, prevention, and clinical signs and symptoms; only 33 (9%) had high knowledge scores (10–18), while 70% thought one virus caused all forms of STDs. Also, only 15% of the respondents knew the clinical features of the *Chlamydia* infection, and 18% identified the correct mode of its transmission. Also, older participants with clinical exposure had a higher knowledge score than young and single females, *p* < 0.05. A positive correlation between age and knowledge score was reported, r (354) = 0.339, *p* < 0.0001. The low knowledge scores were associated with marital status, age, and clinical exposure. Practical strategies to minimize literacy toward sexual education and increase the quality of sexual life must be promoted by educators and the academic curriculum

## 1. Introduction

Sexually transmitted diseases (STDs) are a group of infections that spread from one individual to another through sexual contact. Most STDs’ causative agents can be a wide range of microorganisms, such as bacteria, viruses, parasites, yeast, and fungi [1,2]. Human Immunodeficiency Virus (HIV), *Chlamydia*, Syphilis, Gonorrhea, Genital Herpes Viruses, and Human Papilloma Virus (HPV) are examples of common STDs highly reported in every community [3,4]. Even though STDs affect men and women equally, some STDs might cause more significant symptoms in one gender than in others [5,6]. Most STDs are initially asymptomatic, which increases the risk of transmission to others, while others can cause light symptoms and transient infections or present with pronounced manifestations and serious diseases [7,8,9]. The absence of signs do not exclude STD infection; reproductive health complications such as pelvic inflammatory diseases, infertility, and miscarriage are commonly reported [4,10]. STDs caused by bacteria, such as Gonorrhoea, can be treated by a single dose of antibiotics. However, some STDs caused by viruses, such as genital warts caused by HPV, are not completely curable [11,12]. There are various medications available for the treatment of genital warts, but none have shown to be highly successful in reducing disease transmission. Most are intended to treat surface lesions rather than the illness itself. As a result, there is a relatively high recurrence rate and a need for additional treatment [13,14,15]. In 2018, the WHO documented that more than one million sexually transmitted infections (STIs) were acquired daily. Thus, STDs are a significant worldwide health problem, especially in developing and third-world countries [16]. Moreover, most published data on the prevalence and incidence of STDs come from developed countries [17]. However, some conservative countries’ ethics and social factors cause many data collection obstacles [18]. In traditional societies, where sexual practice is considered taboo and sexual contact before marriage is neither acceptable nor permitted, social pressure prevents the young population from receiving the proper knowledge [19]. According to Madani 2006, some STIs’ prevention measures promoted and implemented in non-Islamic nations are unacceptable in Islamic countries. For example, the notion of “Safe Sex” to prevent STIs, which encourages the use of condoms for sexual encounters regardless the marital status, is strictly forbidden in Islam [20].

Concerns about the rising incidence of sexually transmitted diseases (STDs) among the female community have focused on the well-documented associations between STD knowledge and increased risk of infection at a sexually active age. STDs have considerable social and health consequences, yet little is known about STD knowledge and their implications in the female community [21]. According to Hingson et al., 1990, people’s misconceptions and lack of information about the primary non-HIV STIs put them at greater risk for the repercussions of their sexual conduct [22]. In Sweden, Klanger et al. reported that intercourse appeared to occur sooner in the partnership than ten years ago, and the desire for greater sexual experience had risen. The usage of alcohol during the first intercourse has dramatically reduced. Only 2% had received sex education in school, while 41% of respondents felt they could not discuss sex with their parents [23].

In October 2019, Nguyen et al., reported a lack of knowledge about STIs in Vietnam. They concluded that the cause for the misconception of STDs was an absence of knowledge about these disorders and a lack of sexual health awareness among couples. Almost two-thirds of the study subjects knew that STDs might be treatable, and 34.7% were aware of STD immunizations. Relationships and learning about STDs through the Internet, social networks, and health professionals were all associated with having more awareness about STDs [24]. Moreover, in 2018, Balbeesi and Mohizea reported that 71.1% of respondents said STI indications included genital pruritus, filthy discharge, and dysuria. STIs were thought to be transferred through laying on contaminated bedding, using infected kitchenware, and shaking hands. Moreover, condoms were considered to protect against STIs by almost two-thirds of women. Knowledge of STI transmission routes and symptoms was substantially associated with younger age, greater educational status, and salary [25].

In both the physical and psychological realms, the sexual health of young people is unquestionably a significant issue. The university population in particular is one of the primary groups at risk of infection by STDs [26]. As a result, extra attention must be paid to their STD knowledge to assess probable predictors of preventive activities [27,28]. In Saudi Arabia, an Islamic community with a high appreciation and concern for personal privacy and confidentiality, the sexual health of young people is unquestionably a significant issue, and the university population is one of the primary groups at risk of infection by STDs [29,30]. This sensitivity to STD infection is likely connected to the developmental phase of early adulthood, when sexual experimentation tends to rise [31], as well as various hurdles, such as a lack of knowledge about STDs and difficulty accessing treatment [32].

Although knowledge has been generally excellent and consistent, viral infections and the likelihood of asymptomatic transmission are often inaccurate, resulting in insufficient preventive behavior [33,34]. However, the female sexual knowledge of STDs is not well reported or studied [35]. Even though most STDs are preventable with proper sex education, STDs are still noted among sexually active age groups, such as college students. Therefore, this study aimed to assess the knowledge of STDs among the female college community at KSAU-HS, Jeddah, Saudi Arabia. Our primary objectives were to identify the association between demographic variables and current participants’ knowledge of STDs. Furthermore, this study’s perspective might correct some misconceptions, spread proper knowledge, and convey to health educators the importance of integrating the reality of youth sexuality, awareness, and protection into college curriculums.

## 2. Materials and Methods

### 2.1. Study Design, Setting and Study Period

This cross-sectional study was conducted using a structured questionnaire to assess knowledge of STDs among the adult female college community between October 2019 and November 2020. The female population was either enrolled as full-time students or employees at the female branch of King Saud bin Abdulaziz University for Health Sciences (KSAU-HS)-Jeddah, Saudi Arabia. KSAU-HS is a public university specializing in health sciences and accredited by the Ministry of Education for various undergraduate degrees.

### 2.2. Sample Size and Sampling Technique

Female participants aged 18 to 50 were indiscriminately selected for this study. It was determined that the female population at King Saud bin Abdulaziz University for Health Sciences—Jeddah was about 1815–1539 students and 276 employees. The projected number of staff and students required was determined using the single proportion method, with 50% of the population proportion figure from prior research performed in Saudi Arabia [25]. The sample size was estimated using a cross-sectional research design (n = needed sample size n = Z (/2) 2 pq/d2), which relied on Raosoft, Inc. http://www.raosoft.com/samplesize.html (accessed on 1 October 2019) for the calculation. With 50% predicted knowledge of STDs and a margin of error of 5%, the required sample size was computed at the 95 percent confidence level (CI). The minimum sample size was determined to be 260; the final sample was 325 to account for a 25% non-response rate.

### 2.3. Study Participants and Data Collection Procedures

The sampling technique was a quota sampling method for the selection from all four colleges around the campus: College of Nursing (CON), College of Medicine (COM), College of Applied Medical Sciences (CAMS), and College of Science and Health Professions (COSHP). In accordance with the following table, the total number of students and employees was estimated, and the exact percentages were taken from the required sample size of 276:49, as shown in Table 1.

### 2.4. Instruments of the Study

The structured STD-KQ questionnaire was adopted from Jaworski et al., 2007 and used to collect the participants’ knowledge of STDs [36]. The survey was conducted anonymously, without identifiable data. Participants’ data were collected via an online survey using the Google Forms platform. The questionnaire was distributed among the female population using official emails, and the questionnaire was distributed online in the English language. It was mandatory to complete all primary data variables required in the survey to avoid missing or incomplete data in the analysis. It consisted of two sections and contained several themes: the first section included questions regarding demographic profile, clinical exposure, level of education, type of training, and occupational status; the second section had 27 STD-knowledge questions. The primary outcome variable was the knowledge score, which was taken as the total score and ranged from 0 to 27; the questionnaire consisted of true-or-false questions related to knowledge about STDs, and the score was represented and rated on a three-point categorical scale as low (0–9), medium (10–18), and high (19–27).

### 2.5. Statistical Analysis

Qualitative data were reported as frequency and percentage, whereas continuous variables were provided as mean, standard deviation (S.D) median, minimum, and maximum. The Chi-square test of independence was performed to compare categorical data and analyze the relationship between STD-KQ scores, which were categorized into three levels, and other study variables. The Kruskal–Wallis test, a nonparametric test, was used to compare categorical variables. Logistic regression was applied to investigate the strength of the link between STD knowledge score, marital status, and professional exposure. The significance level was set at <0.05. Collected data were tabulated and statistically analyzed using JMP Statistics for Windows, version 15.

## 3. Results

### Participants Demographic Characteristics

A total of three hundred fifty-five participants responded positively to the dispensed survey and were enrolled in this study. In this study, of the 355 participants, 29 (8.17%) were employees and 326 (91.83%) were students from four professional colleges on the Jeddah campus. Our participants’ median (I.Q.) age was 20 (3). STD knowledge scores, which were assessed using the STD-KQ scale, had a mean (S.D.) score of 8.77 (5.6); 178 respondents (50.14%) received low scores, 144 (40.56%) received average scores, and 33 (9.30%) received high scores in items related to STDs knowledge. This study measured the association between female knowledge about STDs and demographic factors such as age, marital status, clinical exposure, and professional colleges. Table 2 shows a statistically significant association between STD knowledge score and the following variables: age, occupational status, marital status, level of education, clinical exposure, and professional college. Essential study characteristics and associated demographic factors are summarized in Table 2.

Regarding participants’ age, most respondents were younger than 25 (324, 89.85%). Thus, this study reported a significant association between female age and the total score; logistic regression analysis revealed a statistically significant, moderate positive correlation between age and total knowledge score, r (354) = 0.339, *p* < 0.0001. A total of 21 (6.48%) participants who were older than 25 years reported moderate to high scores, compared to 134 (41.36%) younger participants who received moderate scores. Regarding the association between female knowledge about STDs and occupational status, there is a significant difference in participants’ mean scores based on their occupational status, χ2 (2) = 9.1, *p*-value < 0.00106 A total of 19 (65.51%) employed participants received moderate to high scores, compared to 157 (48.15%) students.

In addition, the association between female knowledge of STDs and marital status shows a significant difference in mean scores between single and married, χ2 (3) = 1.534 × 10^−8^, *p* < 0.0001. Most of the participants in our sample were single—330 (92.96%)—compared to married and divorced, which numbered twenty-two (6.2%) and three (0.85%) out of the overall sample, respectively. This study reported that 18 (81.8%) married females had moderate to higher knowledge scores compared to 155 (46.96%) that were single. Regarding the association between female knowledge about STDs and their educational level, χ2 (3) = 21.22, *p* <0.0001. Of the participants who had a college and above-college academic level, 15 (62.5%) scored higher than those who received less education.

Additionally, clinical exposure showed that the majority of our students, 142 (40%), were identified as non-clinical, 103 (29.01%) were pre-clinical, and 81 (22.82%) were clinical. Regarding the association between female knowledge about STDs and clinical exposure, there is a significant difference in participants’ mean scores based on their clinical orientation, χ2 (3) = 74.176, *p*-value < 0.0001. A total of 69 (71.87%) of the clinical exposure participants received moderate to high scores, compared to 27 (28.13%) with low knowledge scores. Regarding the association between female knowledge about STDs and their professional college, χ2 (3) = 42.53.18, *p* < 0.0001. The following categories were identified for participants: 96 (27.04%) students were from COSHP-J, 120 (33.8%) students were from COM-J, 58 (16.34%) students were from CAMS-J, and 52 (14.65%) students were from CON-J. Thus, participants from the college of medicine reported the highest scores, followed by the college of applied medical sciences, as shown in Table 3.

Logistic regression was used to analyze the relationship between marital status, clinical exposure, and knowledge score for the female community knowledge of STDs (Low (0–9), Moderate (10–18), and High (19–27)); Table 3 shows the analysis for a total of 355 respondents. Goodness-of-fit statistics were utilized to determine whether the model adequately described the data. Since the model is significant (<0.001), there is a significant improvement in fit, as compared to the null model. Hence, the model shows a good fit. The difference between Intercept Only Model and Final Model should be significant. Also, the insignificant value (0.627) of Goodness of Fit indicates no significant differences in the observed data and fitted (assumed) model. This study showed a 13.8% improvement in the knowledge scores based on the marital status (married) and clinical exposure (clinical), compared to the null model, as shown in Table 4.

Table 5 presents the impact of marital status and clinical exposure on the participant’s knowledge scores. As for the impact of clinical exposure, those participants who had no clinical exposure were more likely to obtain low and moderate scores for STDs knowledge, as compared to those who had clinical exposure, and the difference is significant (<0.001). In terms of marital status, married participants had a lower chance of scoring low on the knowledge scores, and single participants had a higher chance of scoring low compared to divorced ones. However, the difference is insignificant. Moreover, single participants had a lower chance of scoring moderate in their knowledge, compared to those who were divorced. The odds of obtaining a low knowledge score were 31.5 and 15.29 times greater for participants with non-clinical and pre-clinical exposures, respectively, compared to those with clinical exposure. The odds of obtaining a low knowledge score were 2.67 times greater and 0.173 times lower for single and married participants, respectively, compared to divorced participants. The odds of obtaining a moderate knowledge score increased with clinical exposure. This shows that the odds of obtaining a moderate knowledge score decreased if the participants were not divorced.

## 4. Discussion

Understanding knowledge associated with women’s sexual health is critical, especially for the sexually active group, to design needed programs or interventions to increase STI knowledge and promote safe sex behaviors. This study aimed to evaluate female college students and staff. The findings suggested that although most of our population was studying or working in a health profession field with some medical background or clinical exposure, a significant number of participants from all different professions and colleges had scored medium to low in their understanding of STDs.

This study assessed the female community’s STD knowledge and risk factors. Thus, it offers insight into the current STD knowledge of the those of the female community who study or work at KSAU-HS. We reached many research participants thanks to a widely circulated survey, which resulted in 355 people in the entire KSAU-HS- Jeddah female community. This study found that KSAU-HS students and employees with higher knowledge were more likely to be older, married, and exposed to clinical training. The total STD-KQ score ranged from 0 to 27, with a mean of 8.77 (SD = 5.57). The analysis revealed that half of the participants had a low score on most items related to STD knowledge. Most of the participants, 179 (50.4%), received low scores, 143 (40.3%) received medium scores, and 33 (9.3%) received high scores on items related to STD knowledge. Out of 27 questions about STD general knowledge, we report that none of the participants answered all questions correctly. It was reported that only one question was answered correctly by 250 (70%) participants, while nine had the highest correct responses, ranging from 40% to 60%.

On the other hand, there were twelve questions whose correct answers came from only 15% to 30% of the population (Table 3). A total of six questions were focused on general knowledge about different STDs and their causes. A total of 41 (11.6%) participants believed that “the virus causing HPV is also causing HIV,” while 80 (22.5%) did not know that “different microorganisms cause STDs’. Also, 163 (45%) participants did not know that “some serotypes of HPV can cause cervical cancer in women.” Interestingly, 71 (20%) respondents believe that “STDs are more harmful in men,” as shown in Table 3. Regarding STDs’ mode of transmission, only 64 (18%) of the population responded correctly to the method of *Chlamydia* transmission.

Many respondents were unaware that frequent urination could be a symptom of *Chlamydia* infection, and 171 (48.2%) did not know that anal sex increases the risk of acquiring hepatitis B. In addition, 72 (20.3%) participants were not aware that washing the genitals after sexual intercourse can reduce the chances of developing genital warts, as shown in Table 3. In terms of the level of knowledge regarding STD protection and treatment options, a total of 168 (47.3%) and 166 (46.8%) participants thought that “there is no cure for Gonorrhea and *Chlamydia*,” while 90 (25.4%) of the respondent knew “that there is no gonorrhea vaccination.” Almost half of the participants, 203 (57.2%), were unaware of possible gonorrhea re-infection after a previous infection. Only 43 (12.1%) participants knew condoms were not 100% protective against HIV, as shown in Table 3. Regarding clinical features, only 56 (15%) of the respondents knew that clinical characteristics were associated with chlamydia infection. Moreover, 213 (60%) did not know if a woman could tell if she was infected with Gonorrhea by visible signs or symptoms. A total of 92 (25.9%) thought open sores on the genitals were associated with HIV infection. 

The results indicate some basic understanding regarding STDs, where 70% of the participants answered the following question correctly: does the same virus causes all STDs? The study demonstrates a lack of knowledge regarding STD transmission mode, protection, prevention, and standard clinical features (signs and symptoms). For example, only 15% of the respondents knew the clinical features of chlamydia infection. Moreover, 18.03% of the population got the mode of transmission of chlamydia question right. A considerable percentage, 82%, did not know or thought *Chlamydia* could be contracted due to frequent urination. The analysis confirms a lack of awareness regarding protection and prevention methods for STDS. For example, only 12.11% of the participants knew that condoms prevent HIV. In addition, only 29.01% were aware of possible gonorrhea re-infection after a previous infection. Moreover, 25.35% of the respondents know that there was no gonorrhea vaccination.

According to Wafa et al., ”awareness of Sexually Transmitted Diseases among Adolescents in Saudi Arabia”, 292 (60%) knew that condoms are not 100% protective against all STDs. Their results contradicted our findings regarding condom use, where only 12.11% of participants knew that condoms are not protective against HIV [29]. Our results show that despite the respondents’ medical background, there is a deficit in knowledge and education regarding STDs, which is reflected in our data analysis and total score results. In addition, according to a recent study in Saudi Arabia by Hossam et al., Saudi Arabia’s contribution to HIV cases jumped from 28.9% to 43.5% in the previous decade. Due to the societal stigma associated with STDs, the overall number of reported cases of STD monitoring may be significantly underestimated [30]. In an Islamic society where non-marital sexual activity is prohibited, there are non-reported STD cases, which could mean that the prevalence of STDs in Saudi Arabia is higher than reported. Our results suggest that the decreased awareness and education concerning protection from STDs could contribute to the increased prevalence of STDs. Our results contradict the Malaysian study, which reported that approximately one-third of respondents claimed to have learned about STIs via school or college lectures. Even though sexual health was taught in schools, the researchers found that insufficient knowledge was provided [37].

Furthermore, similar studies were conducted in Bangladesh, Saudi Arabia, King Abdul-Aziz University, and England, which asked identical questions to identical age groups [21,30,31]. Our population was limited to female healthcare workers. In contrast, in Europe and Bangladesh, the studies included higher sample sizes, more genders, and varying levels of education, social status, and age. In addition, most of the surveys were conducted for people in non-medical professions, which adds to our data that a lower awareness of STDs is not only among females in the medical profession but also among both genders from non-medical professions. In the same study conducted in Saudi Arabia’s King Abdulaziz university by Wafa et al., they reported that many participants were aware of STD prevention and that 28% of the subjects did not want to know if they had an STD [29]. This raises concern regarding our population, where intervention and education must be used to avoid STIs’ consequences and complications. The results are strictly confined to one gender and population: healthcare professionals. In addition, despite having many age groups, most participants fell between 18 and 25. Moreover, most participants were unmarried—330 out of the 355 reported to be single, and culturally, it is not acceptable for unmarried girls to ask questions regarding sexual behavior and protection. Thus, this may have affected our results, which could have been different if conducted in other populations where cultural restrictions are not applied.

Despite a limited number of participants, this study’s results are valid for answering the research question. The small sample size limits the generalizability of the results. The cross-sectional study design makes drawing conclusions difficult, as questions were distributed among our population through an online survey. Thus, some questions were not thorough but were sufficient to understand the current awareness of STDs among the participants. As an outcome, additional cohort studies should be conducted to determine further information. Also, most participants were young—319 (89.85%) were under 25. As a result, future research should concentrate on the elderly female population.

## 5. Conclusions

Sexually transmitted diseases are preventable; primary prevention can reduce morbidity and severe complications and, therefore, need attention. The present results suggest that the understanding of STIs has decreased marginally compared to prior local surveys, although it is still inadequate. The Internet is the primary source of information on STIs, with HIV being the most well known. There is a lack of knowledge among most medical students and staff in KSAU-HS regarding STDs. Thus, education about sexually transmitted diseases should be incorporated into the college curriculum or schools to reduce infection risk and expand knowledge about the disorders.

## Figures and Tables

**Table 1:** Sampling study participants.

Study Participants Table						
	Students			Employees		
Location	N	n	(%)	N	n	(%)
College of Medicine (COM)	218	39	(14.17)	45	8	(16.3)
College of Science and Health Profession (COSHP)	829	149	(53.87)	91	16	(32.9)
College of Applied Medical Sciences (CAMS	184	33	(11.97)	39	7	(14.13)
College of Nursing (CON)	308	55	(20.01)	101	18	(36.59)
Total	1539	276	(100)	276	49	(100)

**Table 2:** Essential study characteristics and their association with STD knowledge score (n = 355).

			STDs Knowledge Score						
Characteristics	N = 355		Low Score		Moderate Score		High Score		
	n	%	n	%	n	%	n	%	*p*-Value
Age (years)									
25	324	89.85%	169	52.16%	134	41.36%	21	6.48%	<0.0001 *
≥25	31	10.14%	10	32.26%	9	290.3%	12	38.71%	
Occupation status									
Student	326	91.83%	169	47.61%	146	41.13%	11	3.10%	0.0106 *
Employee	29	8.17%	10	34.48%	12	41.38%	7	24.14%	
Marital status									
Single	330	92.96%	175	53.03%	132	40.00%	23	6.97%	<0.0001 *
Married	22	6.20%	4	18.18%	8	36.36%	10	45.45%	
Divorced	3	0.85%	0	0.00%	3	100.00%	0	0.00%	
Educational level									
Post-graduate education	24	6.76%	9	37.50%	13	54.17%	2	8.33%	0.0017 *
College education	209	58.87%	92	44.02%	89	42.58%	28	13.40%	
Secondary education	94	26.48%	63	67.02%	29	30.85%	2	2.13%	
Elementary education	28	7.89%	15	53.57%	12	42.86%	1	3.57%	
Profession colleges									
College of Sciences and health professions	99	27.89%	71	71.72%	26	26.26%	2	2.02%	<0.0001 *
College of applied medical sciences	70	19.72%	39	55.71%	27	38.57%	4	5.71%	
College of medicine	134	37.75%	46	44.23%	64	47.76%	24	17.91%	
College of nursing	52	14.65%	23	44.23%	26	50.00%	3	5.77%	
Clinical exposure									
Clinical	96	27.04%	27	28.13%	42	43.75%	27	28.13%	<0.0001 *
Non-clinical	156	43.94%	104	66.67%	49	31.41%	3	1.92%	
Pre-clinical	103	29.01%	48	46.60%	52	50.49%	3	2.91%	

Asterisk (*) indicates significant.

**Table 3:** Assessment of the female knowledge about different STDs.

Questions	True	False	I Do Not Know
	n (%)	n (%)	n (%)
1 Genital Herpes is caused by the same virus as HIV?	67 (18.9)	180 (50.7)	108 (30.4)
2 Frequent urinary infections can cause Chlamydia?	93 (26.2)	64 (18.0)	198 (55.8)
3 There is a cure for Gonorrhea.	157 (44.2)	30 (8.5)	168 (47.3)
4 It is easier to get HIV if a person has another Sexually Transmitted Disease.	185 (52.1)	68 (19.2)	102 (28.7)
5 Human Papillomavirus (HPV) is caused by the same virus that causes HIV.	41 (11.6)	170 (47.9)	144 (40.6)
6 Having anal sex increase a person’s risk of getting Hepatitis B.	137 (38.6)	47 (13.2)	171 (48.2)
7 Soon after infection with HIV, a person develops open sores on their genitals (penis or vagina).	92 (25.9)	100 (28.2)	163 (45.9)
8 There is a cure for Chlamydia.	161 (45.4)	28 (7.9)	166 (46.8)
9 A woman who has Genital herpes can pass the infection to her baby during childbirth.	196 (55.2)	57 (16.1)	102 (28.7)
10 A woman can look at her body and tell if she has Gonorrhea.	71 (20.0)	71 (20.0)	213 (60.0)
11 The same virus causes all Sexually Transmitted Diseases.	25 (7.0)	250 (70.4)	80 (22.5)
12 Human papillomavirus (HPV) can cause Genital Warts.	146 (41.1)	24 (6.8)	185 (52.1)
13 Using a condom can protect a person from getting HIV.	228 (64.2)	43 (12.1)	84 (23.7)
14 Human papillomavirus (HPV) can lead to cancer in women.	169 (47.6)	23 (6.5)	163 (45.9)
15 A man must have vaginal sex to get Genital Warts	53 (14.9)	95 (26.8)	207 (58.3)
16 Sexually Transmitted Diseases can lead to health problems that are usually more serious for men than women.	71 (20.0)	127 (35.8)	157 (44.2)
17 A woman can tell that she has *Chlamydia* if she has a bad-smelling odor from her vagina	153 (43.1)	56 (15.8)	146 (41.1)
18 If a person tests positive for HIV, the test can tell how sick the person will become.	115 (32.4)	117 (33.0)	123 (34.7)
19 A vaccine is available to prevent a person from getting Gonorrhea.	50 (14.1)	90 (25.4)	215 (60.6)
20 A woman can tell by how her body feels if she has a sexually transmitted disease.	124 (34.9)	90 (25.4)	141 (39.7)
21 A person with Genital Herpes must have open sores to give the infection to their sexual partner.	93 (26.2)	88 (24.8)	174 (49.0)
22 There is a vaccine that prevents a person from getting Chlamydia.	42 (11.8)	93 (26.2)	220 (62.0)
23 A man can tell by how his body feels if he has hepatitis B.	87 (24.5)	99 (27.9)	169 (47.6)
24 If a person had Gonorrhea in the past, they are immune (protected) from getting it again.	48 (13.5)	104 (29.3)	203 (57.2)
25 Human Papillomavirus (HPV) can cause HIV.	49 (13.8)	132 (37.2)	174 (49.0)
26 A man can protect himself from getting Genital Warts by washing his genitals after sex.	72 (20.3)	103 (29.0)	180 (50.7)
27 A vaccine can protect a person from getting Hepatitis B.	213 (60.0)	40 (11.3)	102 (28.7)

**Table 4:** Knowledge score prediction based on marital status and clinical exposure.

Case Processing Summary			
		N	Marginal Percentage
Total score moderate score	Low	179	50.4%
	Moderate	143	40.3%
	High	33	9.3%
Level of training	Non-Clinical	156	43.9%
	Pre-Clinical	103	29.0%
	Clinical	96	27.0%
Marital status	Single	330	93.0%
	Married	22	6.2%
	Divorced	3	0.8%
Total		355	100.0%

**Table 5:** The impact of marital status and clinical exposure on the participant’s knowledge scores.

Parameter Estimates									
Total Score Moderate Score ^a^		B	Std. Error	Wald	df	Sig.	Exp(B)	95% Confidence Interval for Exp(B)	
								Lower	Upper
Low	Intercept	−0.635	3427.911	0.000	1	1.000			
	[Level of Training = 1]	3.451	0.662	27.146	1	<0.001	31.534	8.609	115.501
	[Level of Training = 2]	2.727	0.675	16.338	1	<0.001	15.287	4.074	57.359
	[Level of Training = 3]	0 ^b^			0				
	[Marital Status = 1]	0.982	3427.911	0.000	1	1.000	2.669	0.000	.^c^
	[Marital Status = 2]	−1.756	3427.911	0.000	1	1.000	0.173	0.000	.^c^
	[Marital Status = 3]	0 ^b^			0				
Moderate	Intercept	17.677	0.567	971.991	1	<0.001			
	[Level of Training = 1]	2.252	0.658	11.726	1	<0.001	9.503	2.619	34.476
	[Level of Training = 2]	2.395	0.659	13.224	1	<0.001	10.973	3.017	39.905
	[Level of Training = 3]	0 ^b^			0				
	[Marital Status = 1]	−16.954	0.593	818.317	1	<0.001	4.335 × 10^−8^	1.357 × 10^−8^	1.385 × 10^−7^
	[Marital Status = 2]	−18.792	0.000		1		6.901 × 10^−9^	6.901 × 10^−9^	6.901 × 10^−9^
	[Marital Status = 3]	0 ^b^			0				

a. The reference category is: High. b. This parameter is set to zero because it is redundant. c. Floating point overflow occurred while computing this statistic. Its value is therefore set to system missing.

## Data Availability

The data used to support the findings of this study have been deposited in the 4TU.ResearchData repository [10.4121/21670817].
